# Myosin XI coordinates ABA-induced stomatal closure via microtubule stability and ROS synthesis in drought-stressed *Arabidopsis*

**DOI:** 10.1007/s00299-025-03538-2

**Published:** 2025-06-19

**Authors:** Haiyang Liu, Motoki Tominaga

**Affiliations:** 1 Graduate School of Science and Engineering, Waseda University, 2-2 Wakamatsu-cho, Shinjuku-ku, Tokyo, 162-0056 Japan; 2 Faculty of Education and Integrated Arts and Sciences, Waseda University, 2-2 Wakamatsu-cho, Shinjuku-ku, Tokyo, 162-0056 Japan

**Keywords:** Abscisic acid response, Drought, Myosin XI, Stomatal closure

## Abstract

**Key message:**

**Myosin XI contributes to ABA-triggered stomatal closure via reactive oxygen species signaling and microtubule remodeling, boosting drought tolerance in Arabidopsis.**.

**Abstract:**

ABA is a key hormone induced by drought stress, and it can regulate stomatal closure through the homeostasis of reactive oxygen species (ROS) and microtubule depolymerization in guard cells, which ultimately enhances plant drought resistance. In this study, we found that myosin XI double (*2ko*) and triple (*3ko*) mutants not only exhibited reduced drought resistance but also showed a weakened ABA response compared to the wild-type (WT). Through comprehensive phenotypic analysis and cellular observations, our experiments demonstrated that myosin XI plays a role in regulating ABA-induced ROS synthesis and microtubule depolymerization in guard cells, thereby facilitating stomatal closure, which minimizes leaf water loss while enhancing drought tolerance in *Arabidopsis*. This study provides novel insights into the role of myosin XI in abiotic stress responses by showing connections with fundamental ABA signaling pathways and broadens our understanding of myosin XI function in plants beyond its established roles in cytoplasmic streaming.

**Supplementary Information:**

The online version contains supplementary material available at 10.1007/s00299-025-03538-2.

## Introduction

Drought is a serious threat to crop yield and quality. Over the past decade, drought-induced agricultural losses have surpassed $30 billion globally (Gupta et al. 2020), a challenge further intensified by climate change. As sessile organisms, plants cannot escape adverse environmental fluctuations (Fang and Xiong 2015). Instead, they have evolved intricate regulatory mechanisms to counteract abiotic stress. These mechanisms encompass diverse physiological and molecular adaptations spanning multiple biological scales (Agurla et al. 2018; Gupta et al. 2020; He et al. 2024).

Abscisic acid (ABA) is a hormone that acts as a central regulator of plant adaptive responses. Under favorable conditions, ABA levels remain low, supporting plant growth. However, during abiotic stress, ABA rapidly accumulates, activating stress-responsive mechanisms that balance defense responses and growth (Chen et al. 2020). In soybean, a genome-wide association study revealed that ABA regulates more than 50% of genes, either by inducing or suppressing their transcription. It has been suggested that ABA regulates plant growth by inducing genes encoding phospholipases, kinases, and other signaling proteins, while suppressing genes involved in photosynthesis, guard cells, and stomatal conductance (Hirayama and Shinozaki 2007; Yoshida et al. 2014). Among these, stomatal dynamics is an important response for controlling water within the plant (Agurla et al. 2018; Matsuda et al. 2016). ABA mediates stomatal closure by modulating ion channel activity and inhibiting light-mediated H⁺-ATPase activation via C-terminal threonine dephosphorylation. This process involves secondary messengers such as reactive oxygen species (ROS), phosphatidic acid, nitric oxide, hydrogen sulfide, and calcium ions (Inoue and Kinoshita 2017). Additionally, ABA signaling regulates stomatal aperture through cortical microtubules (cMTs) polymerization and depolymerization (Dou et al. 2021; Wang et al. 2023). In guard cells, cMTs display dynamic reorganization during stomatal movement (Eisinger et al. 2012). This process is primarily regulated by the E3 ligase MICROTUBULE-RELATED E3 LIGASE 57 (MREL57) via the ubiquitination and degradation of WAVE-DAMPENED2-LIKE7 (WDL7), a process enhanced by ABA (Dou et al. 2021). Furthermore, recent findings indicate that elevated ABA levels activate SUCROSE NON-FERMENTING-1-RELATED PROTEIN KINASE 2.2/2.3/2.6 (SnRK2.2/2.3/2.6), which phosphorylates SPEECHLESS (SPCH) at serine 240/271, leading to SPCH degradation and suppression of stomatal development (Yang et al. 2022). These findings suggest that ABA signaling not only regulates stomatal closure but also influences its development.

In plant cells, the transport, positioning, and interaction of organelles and vesicles are crucial for cytoplasmic organization and cellular function. Previous studies have suggested that some of these processes are regulated by the actin-myosin XI system (Duan and Tominaga 2018). *Arabidopsis* possesses 13 distinct myosin XI isoforms (Reddy and Day 2001), among which XI-K, XI-2, and XI-1 are major motive forces of cytoplasmic streaming. Several studies have consistently demonstrated that multiple knockouts of these myosin XIs exhibit severely impaired cytoplasmic streaming, resulting in reduced plant growth (Peremyslov et al. 2010; Tominaga and Ito 2015; Veerabagu et al. 2020). Several studies have also suggested the involvement of myosin XIs with abiotic stress responses in plants (Chakrabarti et al. 2022; Hajibarat et al. 2022). In potato (*Solanum tuberosum* L.), gene expression analysis revealed that *StMyoXI-B*, a homolog of *AtXI-K*, is highly expressed under abiotic stress. Additionally, several cis-regulatory elements—including MYB, G-box, ABA-responsive element (ABRE), jasmonic acid (JA), and salicylic acid (SA) motifs—were identified within the myosin XI promoter sequences, highlighting their role in abiotic stress response (Hajibarat et al. 2022). In tall fescue (*Festuca arundinacea* Schreb.), differential gene expression analysis (DEGs) under water-deficit conditions revealed that class XI myosin genes were differentially expressed in the crown, suggesting their role in protecting meristematic tissues during drought stress (Chakrabarti et al. 2022). These findings indicate that myosin XI is important in abiotic stress responses; however, the precise physiological mechanisms remain unclear.

In this study, we demonstrated that myosin XI gene knockouts result in drought-sensitive phenotypes in *Arabidopsis*, primarily due to the reduced ABA sensitivity. This impairment inhibits ABA-induced ROS accumulation and microtubule depolymerization, thereby disrupting stomatal closure and increasing water loss. Our findings reveal, for the first time, the critical role of myosin XI in the ABA regulatory network and abiotic stress responses, providing valuable insights into myosin XI function in plants.

## Materials and methods

### Plasmid construction

To visualize the microtubule in planta, we first amplified the smRSGFP-TUB6 sequence via PCR from pBI121/35S: smRSGFP-TUB6, provided by Dr. Takehide Kato (Nara Institute of Science and Technology). Amplification was performed using the following primers 5′-CACCATGAGTAAAGGAGAAGAACTTTTC-3′ and 5′-TCACTCATGATCCAATATCTCTTCTTC-3′. The PCR product was then subcloned into pENTR/D-TOPO using the pENTR/D-TOPO Cloning Kit (Thermo Fisher Scientific, MA, USA). Finally, smRSGFP-TUB6 in pENTR/D-TOPO was inserted into the Gateway binary vector pGWB502 via Gateway cloning (Thermo Fisher Scientific) following XhoI digestion (Takara Bio, Kyoto, Japan).

### Plant materials

The *Arabidopsis thaliana Col-0* used in this study originated from our laboratory stock. The mutant lines included three previously reported myosin XI T-DNA insertional alleles: *atxi-k* (SALK_067972), *atxi-2* (SALK_055785), and *atxi-1* (SALK_019031) (Peremyslov et al. 2008). The double knockout (*xi-k xi-2*/*2ko*) and triple knockout (*xi-k xi-2 xi-1*/*3ko*) lines for myosin XI were provided by Dr. Valerian V. Dolja (Oregon State University) as described in previous studies (Peremyslov et al. 2010; Prokhnevsky et al. 2008). To generate 35S: smRSGFP-TUB6/WT and 35S: smRSGFP-TUB6/*3ko* lines, we performed *Agrobacterium tumefaciens* (*GV3101*)-mediated in planta transformation, followed by selection with 20 μM hygromycin. Fluorescence microscopy was subsequently employed to confirm GFP expression, and only homozygous lines were used in this study.

### Drought treatment

Following stratification at 4 °C for 48 h, seeds of different genotypes were initially cultivated in small pots (6 cm × 6 cm × 5.5 cm) for one week. Then seedlings were transferred to final growth pots (32 cm × 23 cm × 52 cm) and maintained under long photoperiods (16 h light/8 h dark) for an additional week to ensure normal development. Watering was then discontinued, and images were captured at regular intervals. Once the relative soil water content dropped to 3%–5%, watering was resumed to facilitate recovery, and surviving plants were quantified after three days.

For plate-based treatments, *Arabidopsis* seeds were surface-sterilized through sequential treatments: three 1-min immersions in 75% ethanol (v/v), followed by two 5-min washes in 1% NaClO (v/v) and three final rinses with ultrapure water. Germination assays were conducted by establishing seeds on 1/2 MS medium ± 30% polyethylene glycol 6000 (PEG 6000), maintained horizontally in the growth chamber (long photoperiod), and photographs were taken on day eight for statistical analysis. To evaluate root length, seeds were sown on 1/2 MS medium and cultivated vertically for five days. After that, seedlings were transplanted onto osmotic stress medium and grown under long photoperiod for an additional eight days, and root lengths were quantified using ImageJ software (http://rsb.info.nih.gov/ij/).

### Measurement of water loss

For the water loss assay, rosette leaves from 4-week-old plants of different genotypes were excised, immediately weighed, and placed on filter paper at room temperature (25 °C). Leaf weights were recorded at predetermined intervals over a 12-h period to monitor dehydration. The percentage of water loss was calculated using the formula [(initial weight − final weight)/ (initial weight)] × 100.

### RNA isolation and qRT‑PCR

RNA isolation from differentially treated seedlings was conducted with RNAiso Plus (Takara Bio), followed by qRT‑PCR using StepOnePlus system (Applied BioSystems, MA, USA). Amplification reactions employed UBIQUITIN 10 (UBQ10) for transcript normalization, with primer sequences detailed in Supplementary Table S1.

### Measurements of physiological index

For physiological measurements after drought, *Arabidopsis* shoot tissues were collected following a 14-day treatment. Malondialdehyde (MDA), proline, and chlorophyll content were determined with established protocols (Chen et al. 2024; Yu et al. 2023). Photosystem II functionality was assessed via Photon Systems Instruments (FluorCam 800MF, Brno, Czech Republic) through Fv/Fm analysis.

### Measurement of ROS production

Histochemical localization of hydrogen peroxide was conducted using 3,3′-diaminobenzidine (DAB) staining. Roots from 7-day-old WT and mutant plants, maintained under long photoperiods, were collected post-treatment and subjected to DAB infiltration (1 mg mL⁻^1^, pH 5.8; Nacalai Tesque, Kyoto, Japan). The reaction proceeded under dark condition at 28 °C for 3 h. Following incubation, roots were rinsed with 75% ethanol to remove excess stain, and images were captured under white light for further analysis. Similarly, in-situ detection of O_2_^−^ was performed using Nitrotetrazolium blue chloride (NBT) staining. Roots were submerged in NBT solution (0.5 mg mL^−1^, pH 7.6; Tokyo Chemical Industry, Tokyo, Japan) and incubated at 28 °C for 3 h in the dark. After incubation, roots were rinsed with 75% ethanol to eliminate excess stain, and images were captured under white light for subsequent analysis.

2',7'-dichlorodihydrofluorescein diacetate (H₂DCF-DA) staining assay (MedChemExpress, NJ, USA) was employed to visualize H₂O₂ generation in guard cells, following a previously established method (Zhou et al. 2013). Fluorescence signals in guard cells from epidermal peels were visualized using a confocal laser scanning microscope (FV3000; Olympus, Tokyo, Japan). Fluorescence intensity was quantified using ImageJ. For each sample, approximately 14 images were analyzed, and the experiment was independently repeated three times.

### Stomatal aperture assay

Stomatal aperture measurements were conducted according to a previously established method (Lawrence et al. 2018). 2-week-old plants’ leaves of various genotypes were collected. The lower epidermis of the leaves was peeled off using clear scotch tape, and stomatal apertures were imaged using an All-in-One Fluorescence Microscope (BZ-X710; KEYENCE, Osaka, Japan). To assess stomatal aperture response to ABA, rosette leaves were pretreated with a stomatal opening buffer (50 mM KCl, 0.1 mM CaCl₂, 10 mM MES, and 1% sucrose, pH 6.15) for 2 h before imaging. Leaves were then treated with the same solution supplemented with 5 μM ABA for 30 min. Stomatal aperture analysis was performed using ImageJ.

### ABA sensitivity assay

For ABA sensitivity assays, seeds were surface-sterilized and sown on 1/2 MS medium supplemented with varying concentrations of ABA. After stratification at 4 °C for 48 h, plates were positioned horizontally in a growth chamber under long photoperiods. Germination was assessed after nine days of cultivation. For aerial observation, seeds were initially sown on 1/2 MS medium for five days, and then transplanted onto the medium containing different ABA concentrations and cultured under long photoperiods for an additional seven days. Photographs were taken, and plant materials were collected for chlorophyll content analysis. To evaluate root length, the seedlings treated with the same method as that used in the germination rate experiment, with the exception that seedlings were cultivated vertically in the growth chamber. Then the root length was measured using ImageJ.

### Cortical microtubule measurement in guard cells

cMTs in guard cells were observed following the method described in a previous study (Li et al. 2024). cMTs in the guard cells of 35S:smRSGFP-TUB6/WT and 35S: smRSGFP-TUB6/*3ko* lines were visualized using a confocal laser scanning microscope (FV3000; Olympus). GFP signals in the lower epidermal stomata of the leaves were captured using a 100 × oil-immersion objective, and images were acquired for further analysis.

### Statistical analyses

Statistical significance of group mean differences was assessed using two-tailed Student’s t-tests (*p < 0.05, **p < 0.01, ***p < 0.001) in GraphPad Prism 9.0. For comparisons involving multiple experimental conditions against a control group, one-way ANOVA with Dunnett’s post-hoc analysis was performed (*p < 0.05, **p < 0.01, ***p < 0.001).

## Results

### Multiple myosin XI mutants increased sensitivity to drought stress

**Fig. 1:**
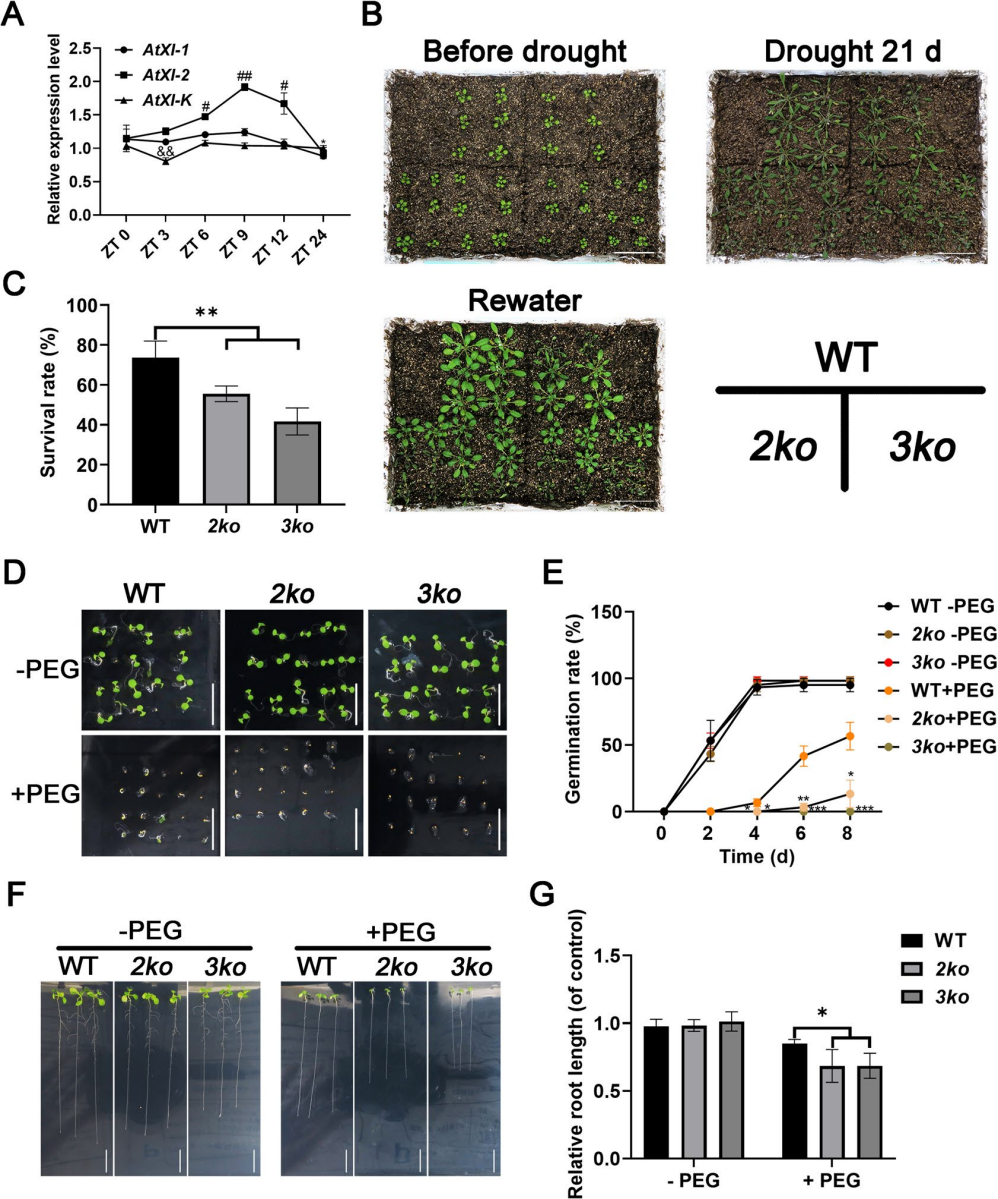
Role of Myosin XI in the drought stress response. **A** qRT-PCR analysis of *AtXI-K*, *AtXI-2*, and *AtXI-1* expression in WT over time following PEG 6000 treatment. Data represent mean ± SD (n = 3; *p < 0.05, **p < 0.01). **B** Phenotypes of WT, *2ko*, and *3ko* plants at the start of drought treatment, after 21 days of drought, and after 3 days of rewatering. Scale bar, 5 cm. **C** Survival rates of WT, *2ko*, and *3ko* following rewatering. Data represent mean ± SD (n = 4; **p < 0.01). **D** Seed germination of WT, *2ko*, and *3ko* on 1/2 MS medium with or without 30% PEG 6000. Scale bar, 1 cm. **E** Statistical analysis of seed germination rates in WT, *2ko*, and *3ko* grown in 1/2 MS with or without 30% PEG 6000. Data represent mean ± SD (n = 3; *p < 0.05, **p < 0.01, ***p < 0.001). **F** Primary root elongation of WT, *2ko*, and *3ko* seedlings grown for 8 days on 1/2 MS medium with or without 30% PEG 6000. Scale bar, 1 cm. **G** Quantification of primary root elongation in WT, *2ko*, and *3ko* seedlings after 8 days in 1/2 MS with or without 30% PEG 6000. Relative root length (WT, -PEG or + PEG): Actual root length (WT, -PEG or + PEG) / Mean of root length (WT, -PEG); Relative root length (*2ko*, -PEG or + PEG): Actual root length (*2ko*, -PEG or + PEG) / Mean of root length (*2ko*, -PEG); Relative root length (*3ko*, -PEG or + PEG): Actual root length (*3ko*, -PEG or + PEG) / Mean of root length (*3ko*, -PEG). Data represent mean ± SD (n = 4; *p < 0.05)

Upon exposure to abiotic stress, plants rapidly reprogram the transcription of stress-responsive genes to maintain cellular homeostasis and support optimal growth and development. qRT-PCR analysis was performed to investigate the correlation between myosin XI expression and drought stress. Among the 13 myosin XI genes present in Arabidopsis, we selected three members, AtXI-K, AtXI-2, and AtXI-1, which are known to be the driving force of cytoplasmic streaming and exhibit functional redundancy in tissue development and cell morphogenesis (Ojangu et al. 2012). The results showed that *ATXI-2* expression was significantly upregulated under drought stress at 6, 9, and 12 h compared to the control (0 h), whereas *AtXI-K* and *AtXI-1* exhibited relatively stable expression levels, except for a slight decrease in *AtXI-K* at 3 h (Fig. 1A).

Next, we analyzed the drought response of single and multiple knockout mutants of AtXI-K, AtXI-2, and AtXI-1. While *atxi-k*, *atxi-2*, and *atxi-1* displayed phenotypes similar to the wild-type (WT) after 21 days of drought treatment (Fig. S1A, B), the *2ko* and *3ko* mutants showed significantly exacerbated wilting (Fig. 1B). Following rehydration, the survival rate of *2ko* was approximately 55%, whereas *3ko* exhibited a strikingly low survival rate of 42%, both markedly lower than the 73% observed in WT (Fig. 1C).

To further assess drought tolerance, we evaluated seed germination under drought stress by supplementing 1/2 MS medium with 30% polyethylene glycol 6000 (PEG 6000). The germination rates of *2ko* and *3ko* were considerably lower than those of WT, with the rate of *2ko* plummeting to approximately 10% and nearly all *3ko* seeds failing to germinate (Fig. 1D, E). Additionally, we examined relative primary root length in seedlings. Root elongation in *2ko* and *3ko* was significantly inhibited compared to WT in 1/2 MS medium containing 30% PEG 6000 (Fig. 1F, G). Collectively, these findings indicate that the loss of myosin XI function increases sensitivity to drought stress.

### Physiological responses of myosin XI mutants to drought stress

**Fig. 2:**
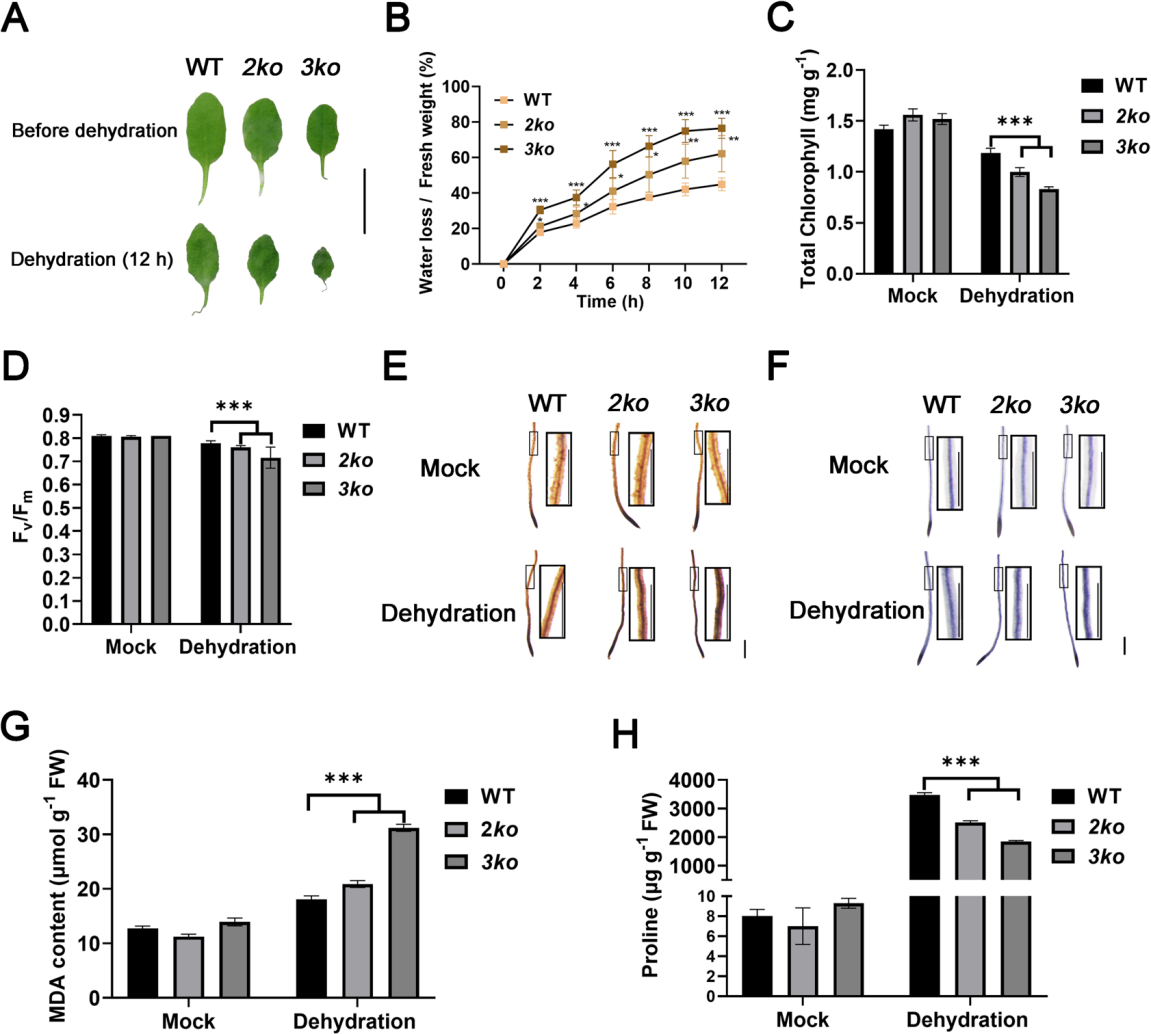
Physiological responses to drought stress. **A** Phenotypic comparison of detached leaves before and after 12 h of dehydration. Scale bar, 1 cm. **B** Water loss from detached leaves of WT, *2ko*, and *3ko*. Data represent mean ± SD (n = 5; *p < 0.05, **p < 0.01, ***p < 0.001). **C** Chlorophyll content in WT, *2ko*, and *3ko* after 14 days of treatment with or without 30% PEG 6000. Data represent mean ± SD (n = 6; ***p < 0.001). **D** Maximal photochemical efficiency of PSII (Fv/Fm) in WT, *2ko*, and *3ko* following 16 days of drought stress. Data represent mean ± SD (n = 10; ***p < 0.001). **E** 3,3ʹ-Diaminobenzidine (DAB) staining of WT, *2ko*, and *3ko* roots after 3 days of treatment with or without 30% PEG 6000. The region enclosed by the black lines presents a magnified view of the area delineated by the square in the original image. Scale bar, 0.5 mm. **F** Nitrotetrazolium blue chloride (NBT) staining of WT, *2ko*, and *3ko* roots after 3 days of treatment with or without 30% PEG 6000. The region enclosed by the black lines presents a magnified view of the area delineated by the square in the original image. Scale bar, 0.5 mm. **G** Malondialdehyde (MDA) content in WT, *2ko*, and *3ko* after 14 days of treatment with or without 30% PEG 6000. Data represent mean ± SD (n = 4; ***p < 0.001). **H** Proline content in WT, *2ko*, and *3ko* after 14 days of treatment with or without 30% PEG 6000. Data represent mean ± SD (n = 7; ***p < 0.001)

Water loss from detached leaves was measured in WT, *2ko*, and *3ko* mutants. After 12 h of detachment, leaves from *2ko* and *3ko* exhibited more pronounced wilting compared to WT (Fig. 2A). The results showed that water loss rate was highest in *3ko* rosette leaves, followed by *2ko*, whereas WT displayed the slowest rate (Fig. 2B). Under drought stress, chlorophyll content significantly decreased in *2ko* and *3ko* compared to WT (Fig. 2C), and both mutants exhibited lower photosynthetic efficiency than WT (Fig. 2D). Drought stress often leads to oxidative damage in plants. To evaluate oxidative stress, we performed DAB staining to detect H_2_O_2_ localization and NBT staining to detect O_2_^−^ in roots. The staining intensity was significantly higher in *2ko* and *3ko* than in WT (Fig. 2E, F), indicating increased oxidative stress in these mutants. Additionally, *2ko* and *3ko* exhibited elevated MDA levels and reduced proline content compared to WT (Fig. 2G, H). These findings suggest that *2ko* and *3ko* mutants experienced significantly greater drought-induced damage than WT.

### Stomatal closure was impaired in myosin XI mutants under drought stress

**Fig. 3:**
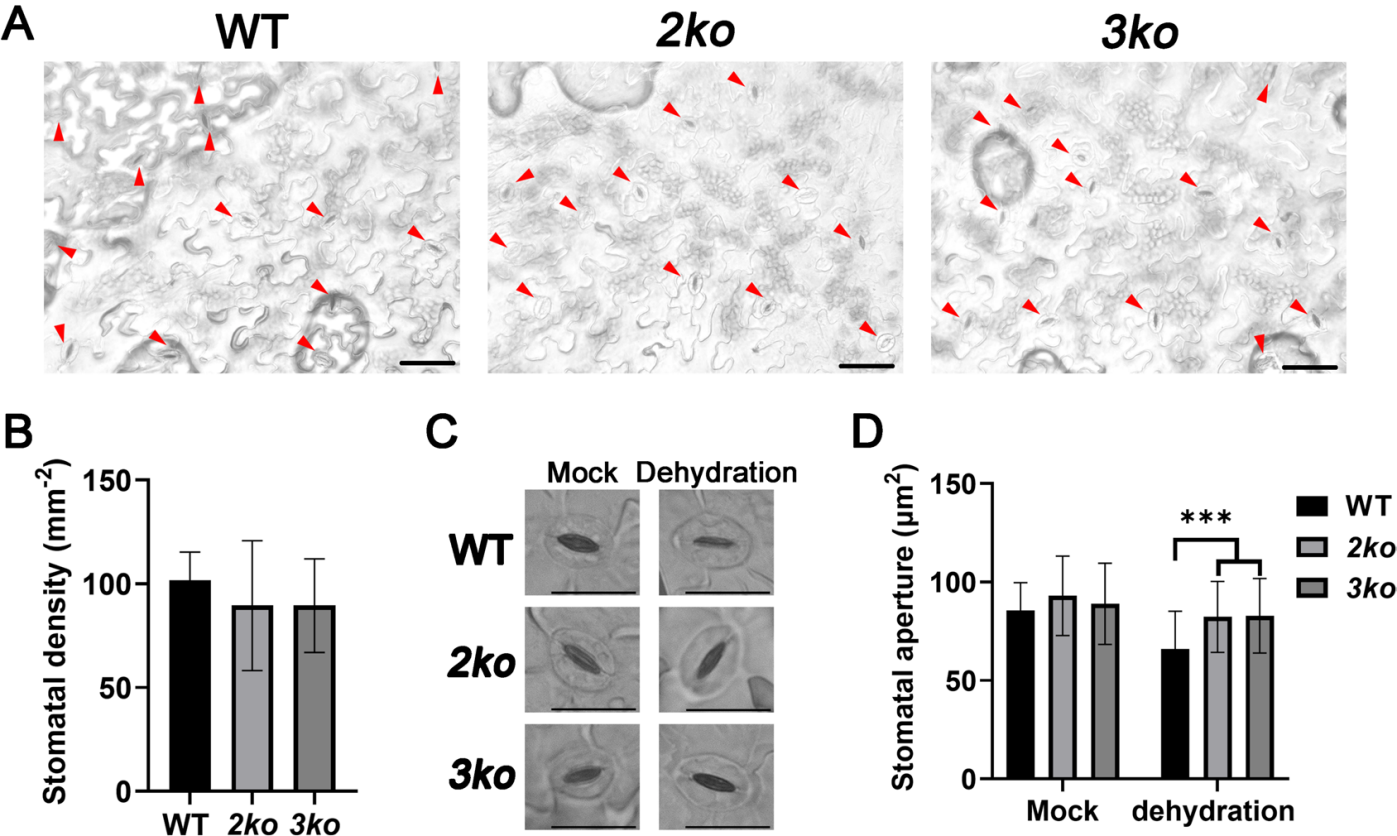
Stomatal responses of WT, *2ko*, and *3ko* to dehydration. **A** Representative image of stomata (red arrowheads) of WT, *2ko*, and *3ko* under normal conditions. Scale bar, 50 μm. **B** Stomatal density in WT, *2ko*, and *3ko* leaves. Data represent mean ± SD (n = 6). **C** Representative images of stomata from detached leaves of WT, *2ko*, and *3ko* were observed immediately or after 2 h of dehydration. Scale bar, 25 μm. **D** Quantification of stomatal apertures from detached leaves of WT, *2ko*, and *3ko* were observed immediately or after 2 h of dehydration. Data represent mean ± SD (n = 40; ***p < 0.001)

Stomatal aperture regulation is a key mechanism by which plants adapt to drought stress. To assess whether myosin XI function influences stomatal traits, stomatal density in the abaxial epidermal layer of WT, *2ko*, and *3ko* leaves was examined under normal growth conditions. The results showed no significant differences in stomatal density among the three genotypes (Fig. 3A, B), indicating that deficiency of myosin XI function does not affect stomatal development. Under normal conditions, stomata were open in WT, *2ko*, and *3ko*, and no notable differences in stomatal apertures were observed among genotypes (Fig. 3C, D). However, after 2 h of dehydration, stomatal apertures decreased in all three genotypes, but stomatal pore size remained significantly wider in *2ko* and *3ko* compared to WT (Fig. 3C, D). These data indicate that myosin XI is involved in facilitating stomatal closure under drought conditions.

### *2ko* and *3ko* mutants exhibit reduced response to abscisic acid

**Fig. 4:**
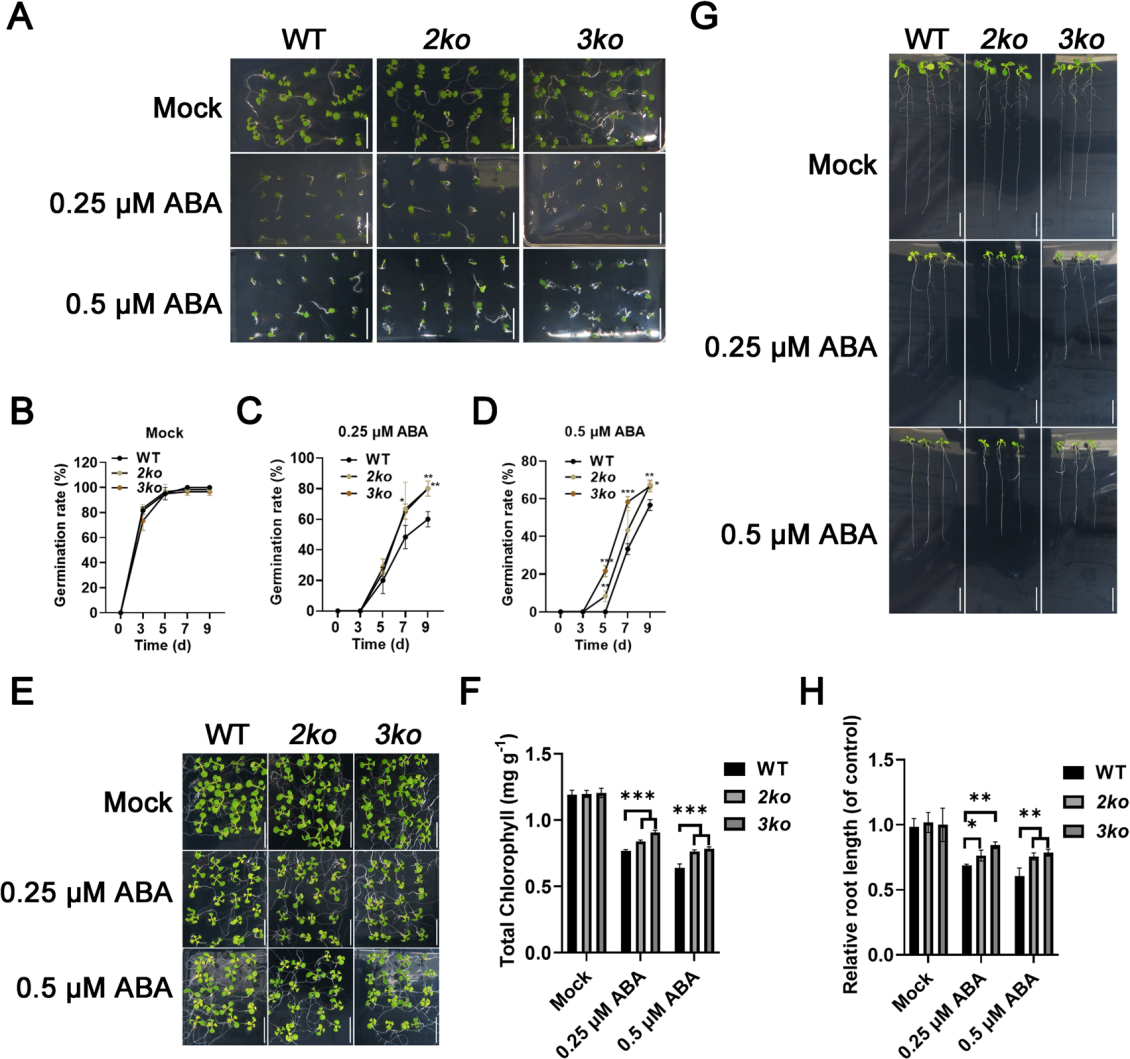
Role of myosin XI in ABA response. **A** Seed germination of WT, *2ko*, and *3ko* on 1/2 MS medium with or without ABA. Scale bar, 1 cm. **B-D** Statistical analysis of seed germination rates of WT, *2ko*, and *3ko* on 1/2 MS without ABA (**B**), with 0.25 μM ABA (**C**), and with 0.5 μM ABA (**D**). Data represent mean ± SD (n = 3; *p < 0.05, **p < 0.01, ***p < 0.001). **E** Aerial phenotypes of WT, *2ko*, and *3ko* grown on 1/2 MS medium with or without ABA for 7 days. Scale bar, 1 cm. **F** Chlorophyll content in the aerial parts of WT, *2ko*, and *3ko* grown on 1/2 MS with or without ABA for 7 days. Data represent mean ± SD (n = 6; ***p < 0.001). **G** Primary root elongation phenotypes of WT, *2ko*, and *3ko* seedlings on 1/2 MS medium with or without ABA for 8 days. Scale bar, 1 cm. **H** Relative primary root elongation of WT, *2ko*, and *3ko* on 1/2 MS with or without ABA for 8 days. Relative root length (WT, -ABA or + ABA): Actual root length (WT, -ABA or + ABA) / Mean of root length (WT, -ABA); Relative root length (*2ko*, -ABA or + ABA): Actual root length (*2ko*, -ABA or + ABA) / Mean of root length (*2ko*, -ABA); Relative root length (*3ko*, -ABA or + ABA): Actual root length (*3ko*, -ABA or + ABA) / Mean of root length (*3ko*, -ABA). Data represent mean ± SD (n = 4; *p < 0.05, **p < 0.01)

The level of sensitivity to ABA serves as a critical determinant in assessing vegetation tolerance to water-deficient conditions (SM 2003). To assess ABA sensitivity, we examined the responses of WT, *2ko*, and *3ko* to ABA treatment. The results showed that *2ko* and *3ko* exhibited significantly higher germination rates than WT when treated with ABA (Fig. 4A, C, D). Under 0.25 μM ABA treatment, germination rates for *2ko* and *3ko* reached 80% by the ninth day, whereas WT only reached 68% (Fig. 4C). Similarly, under 0.5 μM ABA treatment, germination rates for *2ko* and *3ko* were 70%, compared to approximately 50% in WT (Fig. 4D). In contrast, no significant differences in germination rates were observed among WT, *2ko*, and *3ko* on ABA-free medium (Fig. 4A, B). We also evaluated aerial growth in WT, *2ko*, and *3ko*. When treated with ABA, WT exhibited pronounced yellowing and a marked reduction in chlorophyll content compared to *2ko* and *3ko* (Fig. 4E, F). Additionally, analysis of seedlings revealed that when treated with ABA, WT displayed significantly reduced relative primary root length compared to *2ko* and *3ko* (Fig. 4G, H). These findings imply that disruption of myosin XI function leads to diminished responsiveness to exogenous ABA.

### Myosin XIs are involved in the ABA‐mediated stomatal closure

**Fig. 5:**
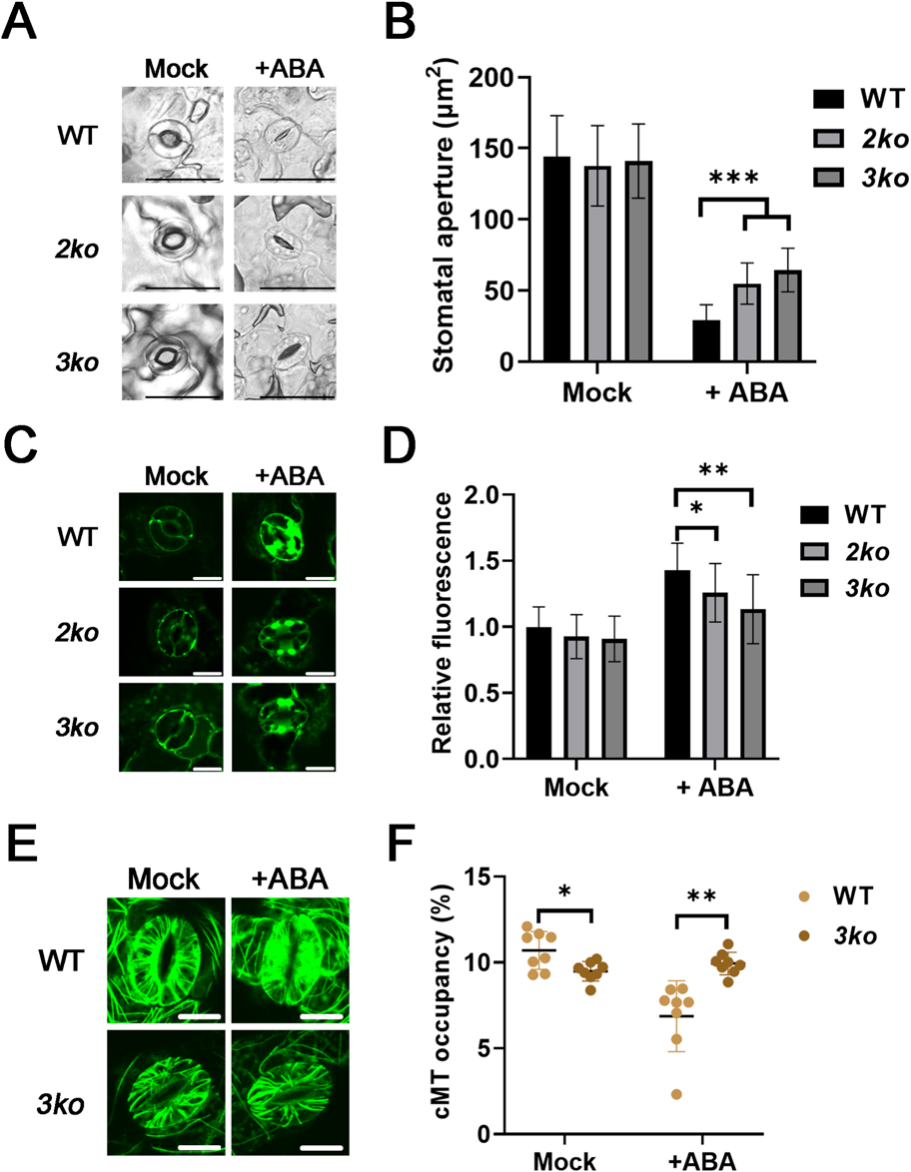
Myosin XI facilitates ABA-induced stomatal closure. **A** Representative images of stomata from detached leaves of WT, *2ko*, and *3ko* after a 2-h treatment with stomatal opening buffer (Mock), followed by a 30-min treatment with ABA (5 μM) (+ ABA). Scale bar, 25 μm. **B** Quantification of stomatal apertures in detached leaves of WT, *2ko*, and *3ko* following a 2-h treatment with stomatal opening buffer (Mock), then a 30-min treatment with ABA (5 μM) (+ ABA). Data represent mean ± SD (n = 40; ***p < 0.001). **C** ROS production in guard cells was detected using the ROS-sensitive fluorescent dye H_2_DCF-DA. Scale bar, 10 μm. **D** Quantification of relative fluorescence in guard cells of WT, *2ko*, and *3ko* plants after a 2-h treatment with stomatal opening buffer (Mock), followed by a 1-h treatment with ABA (30 μM) (+ ABA). Data represent mean ± SD (n = 14; *p < 0.05, **p < 0.01). **E** cMTs labeled with GFP-TUB6 in guard cells of WT and *3ko* were visualized using confocal microscopy. Scale bar, 10 μm. **F** Quantification of cMTs densities in WT and *3ko* following a 2-h treatment with stomatal opening buffer (Mock), then a 1-h treatment with ABA (20 μM) (+ ABA). Data represent mean ± SD (n = 8; *p < 0.05, **p < 0.01)

Given the role of Myosin XI in regulating plant drought resistance, leaf water loss, stomatal aperture during dehydration, and ABA sensitivity (Figs. 1, 2B, 3, 4), we further investigated the stomatal response of WT, *2ko*, and *3ko* to exogenous ABA. After treatment with a stomatal opening solution, stomata from all genotypes fully opened, with no significant differences among them (Fig. 5A, B). However, following ABA application, stomatal pore size in *2ko* and *3ko* remained significantly wider than in WT (Fig. 5A, B), suggesting impaired ABA-induced stomatal closure in the mutants. Water deficit conditions trigger extracellular ROS accumulation in guard cells, which facilitates stomatal aperture reduction via ABA-dependent signaling cascades (Liu et al. 2022). To assess ROS levels in guard cells, we performed H₂DCF-DA staining. The results showed that after ABA treatment, guard cells of WT exhibited higher ROS production than those of *2ko* and *3ko* (Fig. 5C, D), indicating reduced ABA-induced ROS accumulation in the mutants. Since Myosin XI regulates ABA-mediated stomatal closure (Fig. 5A, B), we hypothesized that it might also be involved in ABA-mediated cMTs depolymerization in guard cells. Unexpectedly, in untreated WT guard cells, cMTs exhibited a characteristic radial alignment, whereas in *3ko* plants, this alignment appeared slightly disrupted, with a mild reduction in cMTs density compared to WT (Fig. 5E, F). Following ABA treatment (20 μM for 1 h), filamentous cMTs organization was significantly disrupted in WT guard cells, whereas this effect was markedly less pronounced in *3ko* guard cells (Fig. 5E, F). These results indicate that Myosin XIs contribute to ABA-dependent cMTs depolymerization and ROS generation in stomata, thereby playing a role in limiting transpiration and enhancing drought resistance.

### Myosin XI contributes to the expression of ABA-responsive genes

**Fig. 6:**
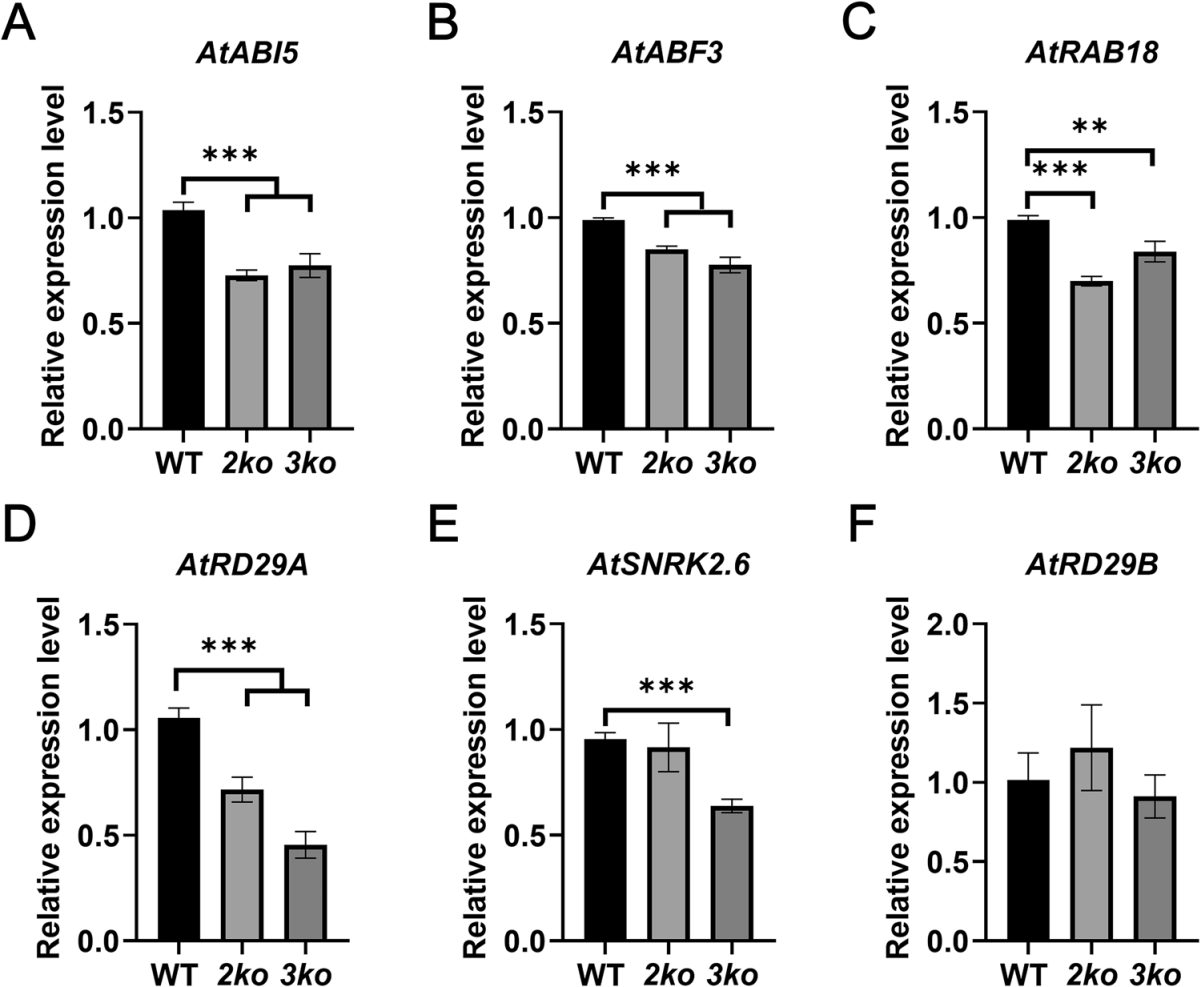
Expression of ABA response-related genes in WT, *2ko*, and *3ko*. **A** Relative expression of *AtABI5*. Data represent mean ± SD (n = 4; ***p < 0.001). **B** Relative expression of *AtABF3*. Data represent mean ± SD (n = 4; ***p < 0.001). **C** Relative expression of *AtRAB18*. Data represent mean ± SD (n = 4; **p < 0.01, ***p < 0.001). **D** Relative expression of *AtRD29A*. Data represent mean ± SD (n = 4; ***p < 0.001). **E** Relative expression of *AtSNRK2.6*. Data represent mean ± SD (n = 4; ***p < 0.001). **F** Relative expression of *AtRD29B*. Data represent mean ± SD (n = 4)

To further examine the involvement of myosin XI in ABA signaling, we quantified transcript levels of ABA-related genes in WT, *2ko*, and *3ko* using qRT-PCR. The results revealed a significant downregulation of multiple ABA response-related genes, including *AtABI5*, *AtABF3*, *AtRD29A*, and *AtRAB18* in both *2ko* and *3ko* compared to WT (Fig. 6A-D). Additionally, *AtSNRK2.6* was specifically downregulated in *3ko* (Fig. 6E), while *AtRD29B* showed no significant changes (Fig. 6F). Notably, the reduction in ABA-related gene expression in *2ko* and *3ko* was not proportional (Fig. 6A, C), suggesting that myosin XI does not directly regulate these genes but may influence their expression through unknown pathways. In conclusion, the loss of myosin XI function leads to reduced expression of specific ABA-responsive genes.

## Discussion

Drought stress triggers various physiological and biochemical responses that influence biomass accumulation and seed productivity in plants. During water scarcity, reducing leaf transpiration is a key strategy to enhance drought resistance. Under drought conditions, stomatal closure is one of the most effective mechanisms by which plants minimize water loss. In this study, we found that myosin XIs contribute to ABA-mediated stomatal closure via cMTs depolymerization and ROS synthesis (Fig. 5). In myosin XI double (*2ko*) and triple (*3ko*) mutants, the expression of ABA response-related genes was reduced (Fig. 6), accompanied by decreased ABA sensitivity (Fig. 4) and increased leaf water loss, ultimately leading to reduced drought resistance in *Arabidopsis* (Figs. 1, 2B).

Under drought stress, stress-responsive genes are rapidly activated to maintain cellular homeostasis. For instance, *SNAC1* in rice (*Oryza sativa*) (Hu et al. 2006), *DREB2A* in maize (*Zea mays*) (Qin et al. 2007), and *LEA25* in wheat (*Triticum aestivum*) (Tunnacliffe and Wise 2007) all undergo rapid transcriptional changes that enhance drought tolerance. In this study, *ATXI-2* expression was significantly upregulated under drought stress, whereas *ATXI-K* and *ATXI-1* transcript levels remained largely unchanged (Fig. 1A). However, the *atxi-2* single mutant exhibited a drought-tolerant phenotype similar to WT (Fig. S1), while drought-sensitive phenotypes were observed only in *2ko* and *3ko* mutants (Fig. 1). These findings suggest that ATXI-2 serves as the primary protein mediating drought stress responses in WT plants. However, in the absence of ATXI-2, its function may be compensated by functionally redundant genes, such as ATXI-K and ATXI-1, facilitating adaptive responses to environmental changes. Further experimental studies are needed to clarify the specific conditions under which this compensation occurs.

ROS are well-known oxidants that, in excessive amounts, can damage proteins, lipids, and nucleic acids. However, numerous studies have shown that ROS play a crucial role in regulating stomatal closure in response to water deficit stress (Postiglione and Muday 2020), primarily through the ABA signaling pathway (Kwak et al. 2003). RESPIRATORY BURST OXIDASE HOMOLOG PROTEIN D/F (RBOHD/RBOHF) function as central mediators of extracellular ROS biosynthesis within stomatal guard cells. In the *rbohD*/*rbohF* double mutant, ABA-induced ROS production and stomatal closure are impaired (Kwak et al. 2003). Additionally, ABA INSENSITIVE1 (ABI1), a downstream regulator in the ABA signaling pathway, also subsequently interacts with RBOHD/F to propel stimulus-responsive ROS biosynthesis (Zhong et al. 2009). Similarly, ABI2 has been shown to interact with GLUTATHIONE PEROXIDASE3 (GPX3) to regulate redox homeostasis in guard cells, thereby modulating stomatal closure (Miao et al. 2006). In this study, *2ko* and *3ko* mutants exhibited insensitivity to ABA (Fig. 4), with significantly reduced ROS production in guard cells compared to the WT following ABA treatment (Fig. 5C, D). These results indicate that myosin XI plays a role in stomatal closure by regulating ROS within the ABA signaling pathway.

cMTs disassembly and rearrangement are important in the regulation of stomatal aperture (Wang et al. 2023). Under optimal water conditions, cMTs adopt a radial arrangement, whereas water scarcity triggers their depolymerization and reorganization in guard cells, facilitating stomatal opening and closure (Yu et al. 2020). ABA regulates stomatal aperture by promoting cMTs depolymerization through the E3 ubiquitin ligase MREL57, which specifically mediates ubiquitination-dependent proteasomal degradation of the microtubule-stabilizing protein WDL7 (Dou et al. 2021). Furthermore, in the ABA-hypersensitive mutant *jul1* (JAV1-ASSOCIATED UBIQUITIN LIGASE1), cMTs retain their radial arrangement following ABA treatment, resulting in open stomata. In this study, ABA treatment led to significantly weaker cMTs depolymerization in *3ko* compared to WT (Fig. 5E, F). This result indicates that myosin XI is involved in ABA-regulated cMTs depolymerization in guard cells and contributes to the regulation of stomatal closure.

A previous study suggests that under drought conditions, roots first detect reduced water availability, rapidly synthesize ABA, and transport it to aerial parts to regulate stomatal closure and activate drought-responsive genes (Kuromori et al. 2018). Based on this, we hypothesize that in multiple mutants, ABA transport may be impaired, leading to delayed activation of ROS signaling and cMTs disassembly. Additionally, in *2ko* and *3ko* mutants, impaired myosin XI function may disrupt the transport of ABA transporters, such as ABCG40 (Kang et al. 2010), or other transcriptional regulators, thereby affecting the expression of ABA-responsive genes (Fig. 6). This disruption further exacerbates the ABA-insensitive phenotype observed in multiple mutants, along with delayed ROS signaling and cMTs disassembly responses. However, the precise mechanism by which myosin XI regulates ROS homeostasis and cMTs disassembly in guard cells via the ABA signaling pathway remains to be elucidated.

In conclusion, this study demonstrates that myosin XI plays a role in ROS synthesis and cMTs depolymerization in guard cells under ABA signaling, thereby regulating stomatal closure. This process reduces leaf transpiration under drought stress and enhances *Arabidopsis* drought resistance. To our knowledge, this is the first study to establish a link between myosin XI and abiotic stress, providing novel insights into myosin XI’s functional role.

## Supplementary Information

Below is the link to the electronic supplementary material.

Supplementary file1 (DOCX 659 KB)

Supplementary file2 (XLSX 13 KB)

## Data Availability

All datasets generated for this study are included in the article/supplemental materials. The data presented in this study are available on request from the first author.
