# Supplementary material for: Myosin XI coordinates ABA-induced stomatal closure via microtubule stability and ROS synthesis in drought-stressed Arabidopsis
Source: Plant Cell Rep. 2025 Jun 19;44(7):147. doi: 10.1007/s00299-025-03538-2 (PMC12179000; doi:10.1007/s00299-025-03538-2)
Supplement: Supplementary file 1 — Supplementary file1 (DOCX 659 KB) [file 299_2025_3538_MOESM1_ESM.docx]

**Plant Cell Reports**

**Myosin XI coordinates ABA-induced stomatal closure via microtubule stability and ROS synthesis in drought-stressed *Arabidopsis***

**Haiyang Liu^1^, Motoki Tominaga^1 2*^**

^1^ Graduate School of Science and Engineering, Waseda University, 2-2 Wakamatsu-cho, Shinjuku-ku, Tokyo 162-0056, Japan

^2^ Faculty of Education and Integrated Arts and Sciences, Waseda University, 2-2 Wakamatsu-cho, Shinjuku-ku, Tokyo 162-0056, Japan

***Corresponding author**

Motoki Tominaga, E-mail, motominaga@waseda.jp; Fax, +81-33-355-0316.


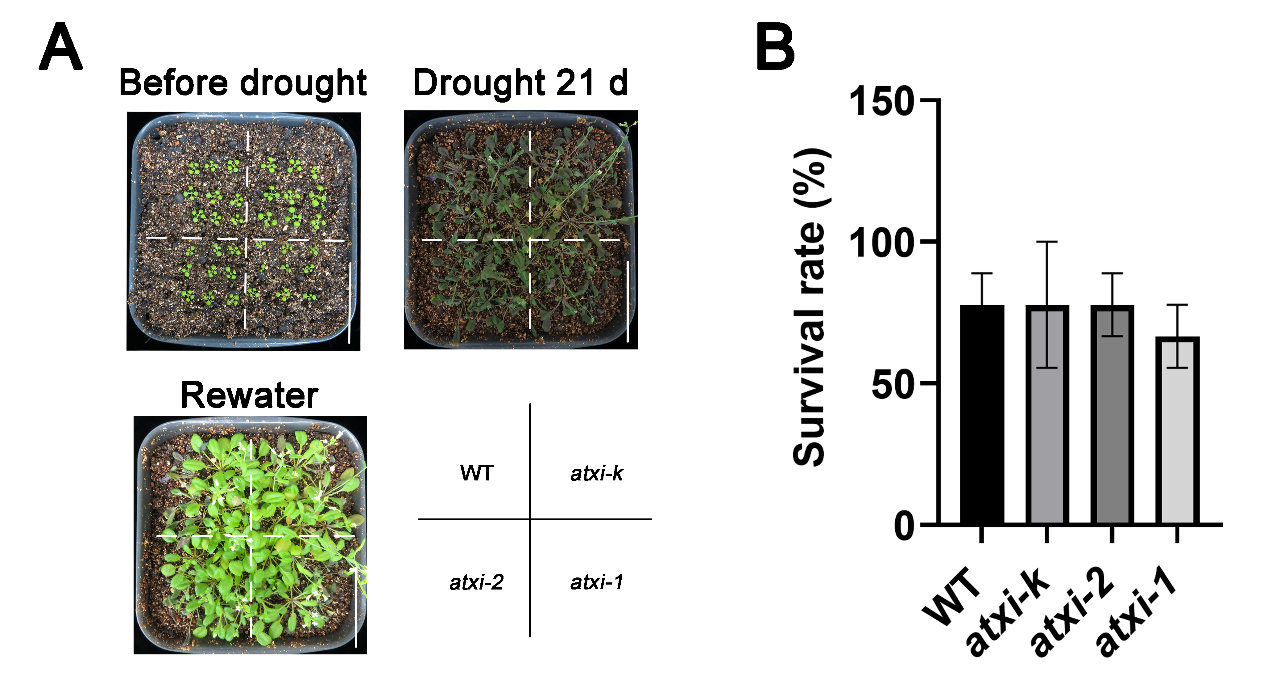


**Fig S1** Role of Myosin XI in drought response. **A** Phenotypes of WT, *atxi-k*, *atxi-2* and *atxi-1* at the start of drought treatment, after 21 days of drought treatment, and after 3 days of rewatering. Scale bar, 5 cm. **B** Survival rates of WT, *atxi-k*, *atxi-2* and *atxi-1* after rewatering. Data represent mean ± SD (n = 3)
